# The Transcriptome Response to Azole Compounds in *Aspergillus fumigatus* Shows Differential Gene Expression across Pathways Essential for Azole Resistance and Cell Survival

**DOI:** 10.3390/jof9080807

**Published:** 2023-07-30

**Authors:** Margriet W. J. Hokken, Jordy P. M. Coolen, Hilbert Steenbreker, Jan Zoll, Tim J. H. Baltussen, Paul E. Verweij, Willem J. G. Melchers

**Affiliations:** 1 Department of Medical Microbiology, Radboud Institute for Molecular Life Sciences, Radboud University Medical Center, 6500 HB Nijmegen, The Netherlandstim.baltussen@radboudumc.nl (T.J.H.B.);; 2 Center of Expertise in Mycology Radboudumc/CWZ, 6500 HB Nijmegen, The Netherlands

**Keywords:** *Aspergillus fumigatus*, azole resistance, isavuconazole, itraconazole, RNA-seq, transcriptome

## Abstract

The opportunistic pathogen *Aspergillus fumigatus* is found on all continents and thrives in soil and agricultural environments. Its ability to readily adapt to novel environments and to produce billions of spores led to the spread of azole-resistant *A. fumigatus* across the globe, posing a threat to many immunocompromised patients, including critically ill patients with severe influenza or COVID-19. In our study, we sought to compare the adaptational response to azoles from *A. fumigatus* isolates that differ in azole susceptibility and genetic background. To gain more insight into how short-term adaptation to stressful azole compounds is managed through gene expression, we conducted an RNA-sequencing study on the response of *A. fumigatus* to itraconazole and the newest clinically approved azole, isavuconazole. We observed many similarities in ergosterol biosynthesis up-regulation across isolates, with the exception of the pan-azole-resistant isolate, which showed very little differential regulation in comparison to other isolates. Additionally, we found differential regulation of membrane efflux transporters, secondary metabolites, iron metabolism, and various stress response and cell signaling mechanisms.

## 1. Introduction

The ascomycete *Aspergillus fumigatus* is a pathogenic fungus that is found across all continents [1,2]. *A. fumigatus* spreads through airborne conidia, which can cause a wide spectrum of diseases in immunocompromised patients depending on the severity of the underlying immunodeficiencies that can result from genetic disorders in both innate and adaptive immune responses, underlying diseases, chemotherapy, or post-transplant immunosuppression [3]. Furthermore, *A. fumigatus* can produce a variety of virulence factors such as gliotoxin, which influence pathogenesis [4]. Lastly, it has been proposed that the pulmonary microbiome influences the severity of infections caused by *A. fumigatus* [5,6].

Additionally, *Aspergillus* infections have been observed among critically ill influenza and COVID-19 patients in the intensive care unit (ICU) [7,8]. Its potential to easily spread across great distances, together with the ability to adapt to novel challenging environments, makes *A. fumigatus* a difficult-to-treat opportunistic pathogen [9,10]. Clinicians remain limited in treatment options; the azoles remain the first choice in the treatment and management of *Aspergillus* diseases. However, the efficacy of the azole class is threatened by the emergence of azole resistance [11].

Azole resistance can develop in patients with a history of azole treatment; however, many resistant *A. fumigatus* isolates are likely to have originated in the environment due to the use of agricultural azole derivatives [1,12,13,14]. Azole class compounds inhibit the sterol 14α-demethylase Cyp51A, an essential enzyme in ergosterol biosynthesis [15]. The majority of resistant isolates harbor a TR_34_/L98H mutation on the *cyp51A* locus, which is highly likely to have an environmental origin due to the use of agricultural azole derivatives and subsequent natural selection of cross-resistant isolates [13]. 

*A. fumigatus* is able to readily adapt to new and stressful environments to maintain growth and reproduction [16,17,18]. Many mutations that enable the fungus to overcome azole pressure have been elucidated in the last decade. These include a wide spectrum of mutations in the *cyp51A* coding area and promotor region and mutations in regulatory genes involved in ergosterol biosynthesis [19,20,21,22,23]. However, not all resistant isolates harbor mutations in *cyp51A* or regulatory genes involved in ergosterol biosynthesis, and isolates can have increased MICs for azole compounds that are not explained by the mutations present in the isolate [24,25]. It has been suggested that several cellular processes ultimately contribute to the resistance profile of an isolate, including increased drug efflux pump activity, mitochondrial dysfunction, and activation of environmental stress response pathways [26,27,28,29]. 

As many of these molecular mechanisms undergo differential regulation of genes involved, transcriptome analysis can give insight into activated mechanisms upon adaptation to azole compounds and shed light on how *A. fumigatus* changes its physiology in order to survive. As individuals can be colonized with susceptible and resistant *A. fumigatus* conidia, we exposed azole-susceptible and resistant isolates to sub-inhibitory concentrations of the azole compounds itraconazole and isavuconazole to monitor changes in gene expression after 48h of azole exposure.

This research shows that several molecular mechanisms are activated upon azole addition to the medium. These findings can shed light on the genetic regulatory networks involved in cellular adaptation to azole compounds, which can enable us to design new antifungal therapies in the future.

## 2. Results

To investigate the transcriptome during exponential growth under antifungal pressure, we conducted an in vitro assay in which we exposed fresh conidia of *A. fumigatus* isolates to sublethal concentrations of itraconazole (ICZ) and isavuconazole (ISA) (Table 1).

The isolates used in this study are listed in Table 1. The mycelial morphology of these isolates grown on MM is shown in Figure S1. STR*af* data show that these isolates are indeed of a separate genotype. These genomic differences underline that the isolates tested in this study have likely originated from a different environment, which supports the assumption that the transcriptome alterations are at least suspected to be different to some extent between these isolates due to genetic differences.

These isolates are either susceptible, resistant to ICZ, or pan-azole-resistant. Mutations on the *cyp51A* locus explain the higher MICs in the latter two isolates, as they contain the well-known TR34/L98H mutation that confers resistance to ICZ, voriconazole (VCZ), and isavuconazole (ISA) and a TR46^3^/Y121F/T289A mutation, which confers resistance to all clinical azoles [30]. The V181(R^PAN^) isolate was originally isolated from a compost heap in The Netherlands [30]. Due to these characteristics, we named these isolates V147(S), V162(R), and V181(R^PAN^).

The transcriptomic response of three strains was compared by inoculating each isolate with IC50 concentrations of ICZ or ISA, as determined by dose–response curves. These isolates were compared to isolates treated with an equal amount of DMSO.

Additionally, we tested the effect of DMSO on untreated samples of susceptible isolate V147(S). All reported log2 fold-change (log2FC) values can be found in Table S3 containing all DEGs after the treatment of these isolates (Table S3).

### 2.1. Exploratory Analysis of DEGs

On average, 18,420,393 uniquely mapped sequence reads were obtained per isolate, ranging from 6,235,921 to 25,617,320 reads per individual sample, corresponding to 32× and 177× genome coverage, respectively. After genome alignment, an average unique mapping percentage of 87.1% to reference genome Af293 was found [31] (Table S2).

**Figure 1:**
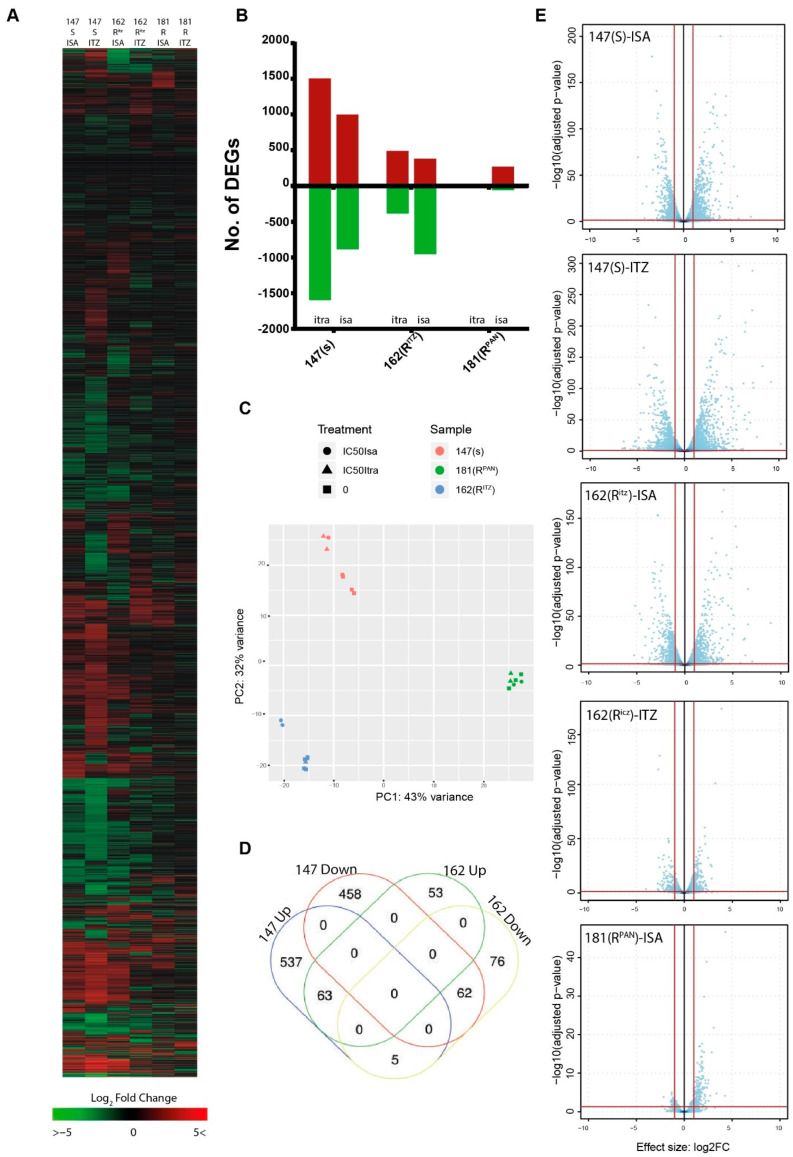
Exploratory results. (**A**). Heatmap of the total transcriptome after hierarchical clustering. (**B**). The total number of DEGs found in each isolate after treatment with ICZ or ISA. (**C**). Principal component analysis shows clustering of biological replicates. The effect of azole treatment on the transcriptome has less influence than the isolate background. (**D**). Overlap of similarly regulated DEGs of isolates V147(S) and V162(R) by Venn analysis. A total of 63 up-regulated core DEGs and 62 down-regulated core DEGs were found. (**E**). Volcano plots of each isolate and treatment. Log2FC values are plotted on the X-axis, whereas the −log10 (adjusted *p*-value) is plotted on the Y-axis. Please note that the Y-axis varies in maximum value when comparing volcano plots.

Heatmap visualization of all differentially expressed genes (DEGs) shows strain-specific transcriptional differences after treatment with ICZ and ISA (Figure 1A). Principle component analysis (PCA) revealed that the transcriptome is highly variable between isolates and that the biological replicates clustered together for each isolate and treatment (Figure 1C). Susceptible isolate V147(S) showed a clear treatment effect for both ICZ and ISA, whereas resistant isolate V162(R) only showed a predominant treatment effect for ISA. Pan-azole-resistant isolate V181(R^PAN^) did not show great variance between treatments and control samples. After ICZ treatment of the isolates, we identified 3102 and 873 DEGs for isolates V147(S) and V162(R), respectively, from a total of 9840 genes (Genome inventory, AF293 genome, AspGD, 1 April 2020) compared to untreated isolates. No DEGs were found for V181(R^PAN^) after ICZ treatment. After ISA treatment of the isolates, we identified 1883, 1795, and 326 DEGs for isolates V147(S), V162(R), and V181(R^PAN^), respectively (Figure 1B). A volcano plot for each time point was created to assess the distribution of the log2FC values relative to the corrected *p*-value (Figure 1E). To investigate if the organic solvent DMSO had any influence on gene expression, we treated susceptible isolate V147(S) with the same volume of DMSO and found 21 DEGs after treatment. The genes that were found were filtered from the list of DEGs retrieved after azole addition to the medium (Table S3). As these results were only available for isolate V147(S), it should be noted that this list might not represent the genetic diversity of all isolates and might not be exhaustive, as this was not compared for isolates V162(R) and V181(R^PAN^).

Lastly, to obtain a better view on which genes are identically regulated in isolates V147(S) and V162(R) during both treatments, we overlapped all DEGs that were similarly regulated during both treatments in a Venn diagram. We found 63 up-regulated core DEGs and 62 down-regulated core DEGs, but the vast majority of DEGs in V147(S) were unique for that isolate (Figure 1D). No significantly enriched FunCat categories or GO terms were found for these gene sets. From all 125 shared core DEGs, only 9 are known and characterized. Among the up-regulated genes for V147(S) and V162(R) were efflux transporter *cdr1B* (Afu1g1433) and thaumatin domain protein *calB* (Afu8g01710), a paralogue of *calA* (Afu3g09690), a cell wall protein that mediates interaction with human integrin β1 and is required for normal virulence [32]. Among the down-regulated genes are siderophore biosynthesis genes SidA (Afu2g07680), and two other siderophore biosynthesis genes, (Afu3g03390) and (Afu3g03400). The other down-regulated core DEGs comprise genes involved in diverse cellular processes such as host–pathogen interaction, cell signaling, and ribosome biosynthesis. All shared DEGs are listed in Table S4. 

Remarkably, when we compared the 268 up-regulated DEGs and 56 down-regulated DEGs from pan-azole-resistant isolate V181(R^PAN^) treated with ISA with these core DEGs, only 12 genes were shared between these isolates, underlining a strong difference in transcriptome adaptation between isolates. Among these genes were a majority of uncharacterized genes and ABC transporters Cdr1B (Afu1g14330) and AbcA (Afu2g15130).

### 2.2. Most DEGs after Azole Treatment

We analyzed the 50 DEGs with the highest positive or negative log2FC per isolate and treatment to assess the individual response of each isolate to different azole compounds (Table 2). The transcript encoding for integral membrane protein Afu2g17930 was the most up-regulated gene with a log2FC of 10.17 in V147(S)-ICZ, 5.37 in V147(S)-ISA, and 5.07 in V162(R) -ISA. This gene is known to be up-regulated upon acute ER stress [33]. It is predicted to play a role in fatty acid hydroxylation and oxidation–reduction processes (AspGD; Prediction of Gene Ontology (GO) annotations based on protein characteristics, 2011) and was also found up-regulated in voriconazole-treated isolates [34]. Several RTA1 domain proteins were present amongst the most up-regulated DEGs, of which Afu6g14140 was the most up-regulated in all isolates. Not much is known about this family of transmembrane proteins in *A. fumigatus*, but their homologs present in *Candida albicans* are also induced by azole compounds and involved in azole resistance through regulation via calcineurin signaling [35,36]. The ABC transporter AbcE (Afu7g00480) was found the most up-regulated in the pan-azole-resistant isolate V181(R^PAN^) with log2FC 4.31 and was moderately up-regulated in isolates V147(S)-ICZ, V147(S)-ISA, and V162(R)-ICZ. Furthermore, we found strong up-regulation of a putative fungal adhesin (Afu2g10130) in V147(S)-ICZ with log2FC 7.3, which was also up-regulated in V147(S)-ISA and V162(R)-ISA. We also found up-regulation of the ‘Aspf4-like’ putative allergen (Afu3g00710) in isolates V162(R)-ICZ and V162(R)-ISA, with log2FC 2.14 and log2FC 3.89, respectively. Additionally, the major allergen Aspf1 (Afu5g02330) was found strongly up-regulated in isolate V147(S)-ICZ with log2FC 5.72 and was moderately up-regulated in V147(S)-ISA and V162(R)-ISA. Several genes present in secondary metabolite gene clusters were found among the most up-regulated genes including *hasC* and *nscA.* Furthermore, genes involved in siderophore biosynthesis and transport, *sidG* (Afu3g03650) and *mirB* (Afu3g03640), are amongst the most down-regulated in isolates V147(S)-ISA, V162(R)-ISA, and V162(R)-ICZ.

### 2.3. Sterol Metabolism and Regulation

The inhibition of Cyp51A by azole compounds resulted in changes in gene expression in the ergosterol biosynthesis pathway, as shown in previous research [30,33]. In this study, we also assessed the expression levels of genes involved in the ergosterol biosynthesis pathway. When we compared our DMSO-treated isolates of V162(R) and V181(R^PAN^) with the susceptible DMSO-treated isolate V147(S), the *cyp51A* expression was log2FC 3.12 higher in isolate V162(R) and log2FC 3.46 higher in V181(R^PAN^), in accordance with the literature as expected [30,37] (Table S6).

**Figure 2:**
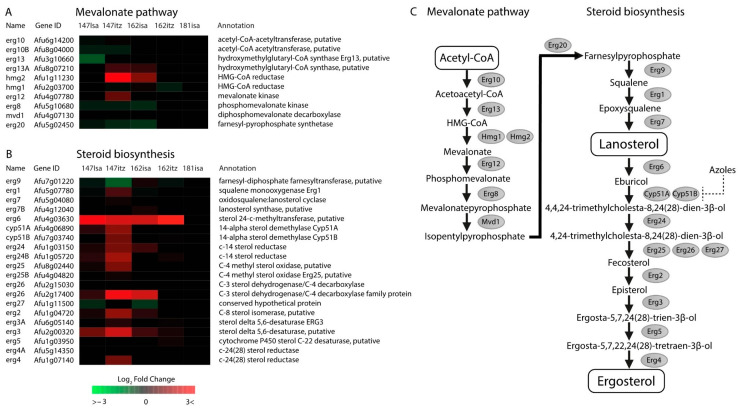
Ergosterol biosynthesis. The colors represent the Log2FC of each time point relative to reference time point t = 0. (**A**). Heatmap of all enzymes with a verified or predicted role in the mevalonate biosynthesis. (**B**). Heatmap of all enzymes with a verified or predicted role in the ergosterol biosynthesis. The *erg6* gene shows strong up-regulation through all isolates and treatments except V180(R^PAN^)-ISA. (**C**). Mevalonate and ergosterol biosynthesis pathways in *A. fumigatus*.

Several genes involved in the biosynthesis of mevalonate, an essential precursor substrate for ergosterol synthesis, are up-regulated in V147(S)-ICZ and V162(R)-ISA (Figure 2). *erg13A* and *hmg2* were found up-regulated in both strains, with the mevalonate kinase *erg12* additionally up-regulated log2FC 1.35 in V147(S)-ICZ. Analysis of ergosterol biosynthesis genes showed up-regulation of the majority of *erg* genes in V147(S)-ICZ, V147(S)-ISA, and V162(R)-ISA (Figure 2). No DEGs were found in V181(R^PAN^) after treatment with ICZ or ISA.

The *cyp51a* eburicol 14α-demethylase and its paralog *cyp51b* were only found to be up-regulated in susceptible isolate V147(S) after both treatments. The *erg6* gene, functioning before Cyp51A in this pathway, was found to be the most up-regulated in both treatments of V147(S) and V162(R). Erg6p was identified as a protein that localizes at lipid droplets in *S. cerevisiae* [37], and a homolog of this protein in *A. nidulans* was found to localize at lipid droplets as well [38,39]. *A. fumigatus* Erg6 shares 88.86% amino acid sequence identity with the *A. nidulans* Erg6 enzyme (NCBI BLASTp). The sterol delta 5;6-desaturase *erg3* (Afu2g00320) was found to be up-regulated in all isolates but the pan-azole-resistant V181(R^PAN^) isolate.

Additional to changes in sterol metabolism, several genes involved in sterol transport and regulation were found to be differentially expressed. In isolates V147(S)-ISA, V147(S)-ICZ, and V162(R)-ISA, we found moderate up-regulation of *oshE* (Afu3g05520), a protein involved in sterol transport, which localizes at contact sites between the ER and the plasma membrane (Table S1) [39]. In isolate V181(R^PAN^)-ISA only, we found up-regulation of putative O-acyltransferase gene Afu2g08380 with log2FC 1.05. This gene is the most homologous to the *S. cerevisiae* ARE2 gene, which is an Acyl-CoA:sterol acyltransferase located in the ER, involved in the esterification of sterols, which prepares them for storage in lipid droplets [40]. Lastly, we found up-regulation of Afu7g01960 in isolates V147(S)-ISA, V147(S)-ICZ, and V162(R)-ISA by log2FC 1.01, log2FC 1.36, and log2FC 1.34, respectively (Table S1). This gene is a putative transcription factor that promotes sterol synthesis and shows 36% amino acid sequence identity with the ‘sterol uptake control protein 2’ UPC2 in *S. cerevisiae* [41].

### 2.4. Siderophore Biosynthesis Pathway Is Down-Regulated

**Figure 3:**
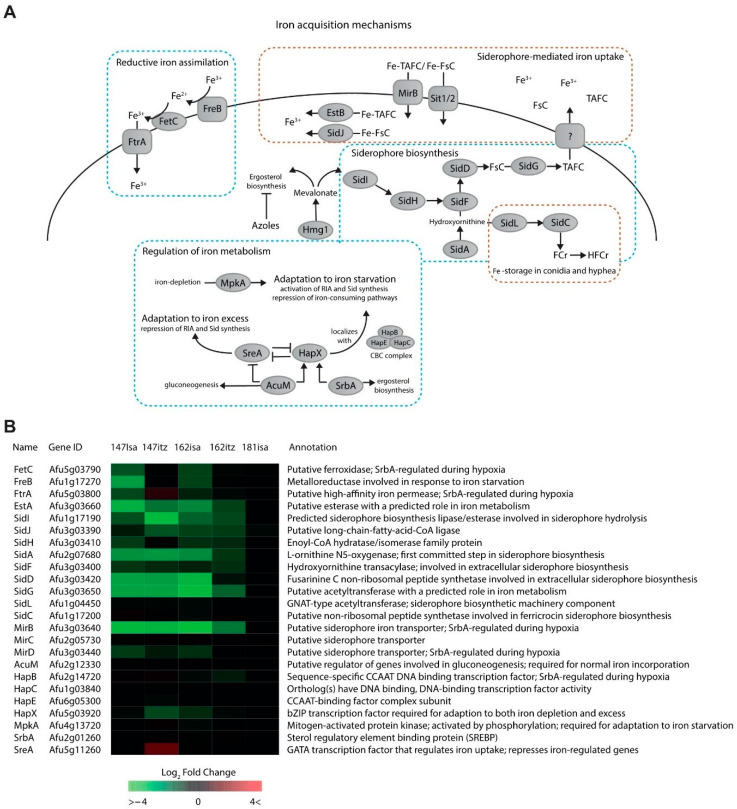
Iron acquisition and siderophore biosynthesis. (**A**). Overview of iron acquisition pathways in *A. fumigatus*. (**B**). Heatmap of genes involved in iron homeostasis.

The siderophore biosynthesis pathway is involved in extracellular iron uptake in low-iron environments and is regulated by key transcription factors SreA and HapX, which are interconnected in a negative feedback loop (Figure 3A) [40,41,42,43]. This pathway shares mevalonate as an important substrate with the ergosterol biosynthesis pathway [44]. Siderophore transporter MirB and the majority of the *sid* genes were down-regulated during both treatments in V147(S) and V162(R) (Figure 3B). Additionally, three genes involved in reductive iron assimilation, *ftrA*, *freB,* and *fetC,* were down-regulated when V147(S) and V162(R) were treated with ISA but not with ICZ. Iron-regulatory transcription factor SreA was found up-regulated by Log2FC 1.67 in isolate V147(S)-ICZ and was only slightly up-regulated in all other isolates. The main transcriptional activator of the siderophore system HapX is found down-regulated during all treatments (Figure 3B).

### 2.5. Membrane Transporter Regulation

The *A. fumigatus* genome contains various transmembrane transporters that are involved in resistance against antifungal compounds [27]. Therefore, we investigated all verified and putative multidrug-efflux transporters identified in *A. fumigatus* and performed hierarchical clustering on these results. 

**Figure 4:**
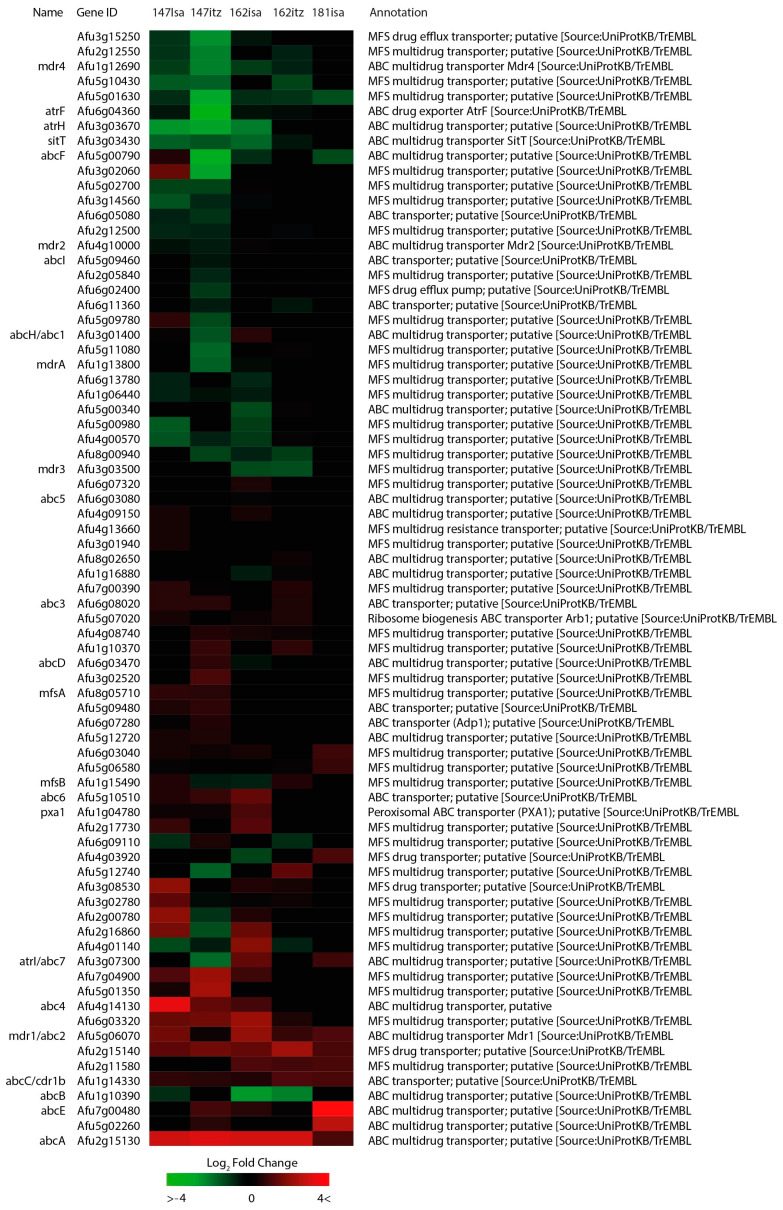
Membrane transporters associated with multidrug efflux. Heatmap showing the hierarchical clustering of the expression patterns of all transporters predicted to play a role in drug efflux in *A. fumigatus*. The colors represent the Log2FC of each time point, relative to reference time point t = 0. Euclidean distances were calculated and complete-linkage hierarchical clustering was performed.

Verified drug-efflux transporters Mdr1, AbcA, and Cdr1B were significantly up-regulated in all isolates treated with ICZ or ISA (Figure 4). Predominantly down-regulated transporters include AbcB, Mdr4, SitT, Mdr3, and AtrF. Surprisingly, the *abcE* gene was found strongly up-regulated by log2FC 4.31 in the pan-azole-resistant isolate V181(R^PAN^), whereas this gene was found to be only moderately up-regulated in other isolates. Clustered together with Cdr1b and Mdr1 are putative drug transporters Afu6g03320, Afu2g15140, and Afu2g11580, which show up-regulation in the majority of isolates. Putative MFS transporter Afu6g03320 was found up-regulated in V147(S)-ISA, V147(S)-ICZ, and V162(R)-ISA by log2FC 1.68, 1.74, and 2.35, respectively. Its homolog in *A. nidulans* encodes a sugar transporter [45]. Putative ABC transporter Afu5g10510 was found up-regulated in V147(S)-ICZ, V147(S)-ISA, and V162(R)-ISA, and its homolog in *A. nidulans* encodes a transporter in the cichorine biosynthesis cluster. In V181(R^PAN^)-ISA only, we found up-regulation of Afu5g02260 with log2FC 2.87, which encodes an ABC transporter homologous to AUS1 and PDR11 in *S. cerevisiae*, two paralogs that facilitate sterol uptake from the environment [46].

### 2.6. Secondary Metabolite Cluster Activation

**Figure 5:**
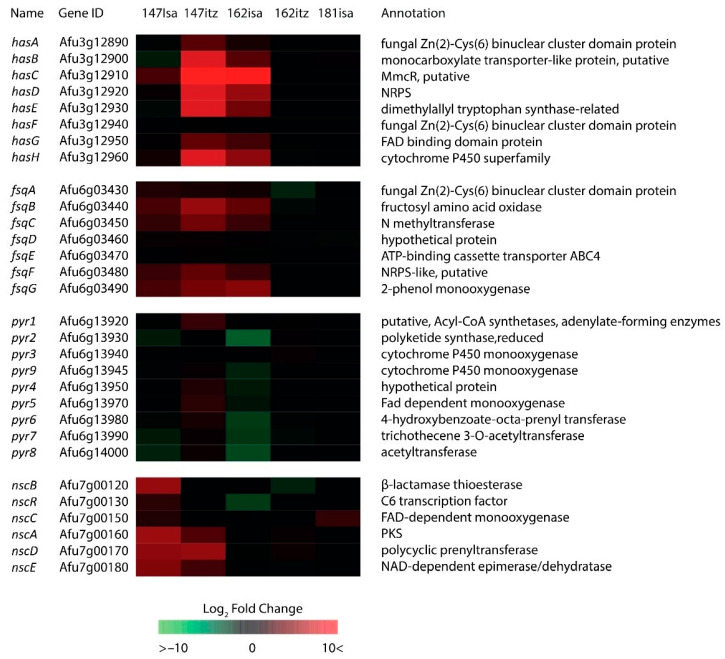
Secondary metabolite production. Heatmap of four secondary metabolite gene clusters that were differently expressed upon addition of ITZ or ISA to the medium.

We assessed the 26 secondary metabolite clusters currently identified in A. fumigatus [47]. Four metabolic clusters show enhanced transcription in our dataset after azole treatment (Figure 5). For an overview of all currently identified secondary metabolite gene clusters in A. fumigatus and their corresponding log2FC values during this experiment, see Table S5.

The *has* cluster consists of eight genes and is involved in hexadehydroastechrome biosynthesis, a molecule with iron-binding properties. An *A. fumigatus* strain with *hasB* overexpression causes a higher mortality rate in a neutropenic murine model [48]. We observed strong up-regulation in the V147(S)-ICZ and V162(R)-ISA isolates. The V147(S)-ISA isolate only showed moderate up-regulation of *hasC, hasD,* and *hasH*, whereas no strong regulation was found in all other isolates. The fumisoquin cluster, which consists of *fsqA-G,* was found up-regulated in V147(S)-ICZ, V147(S)-ISA, and V162(R)-ISA. This cluster produces several plant-like isoquinoline alkaloids which can have a variety of functions [49]. Pyripyropene A was initially discovered as a treatment for hypercholesterolemia and atherosclerosis and was shown to have insecticidal properties [50,51,52]. Interestingly, the pyripyropene A cluster was only found up-regulated in V147(S)-ICZ, whereas it was found down-regulated in V147(S)-ISA and V162(R)-ISA. The neosartoricin/fumicycline A biosynthesis cluster synthesizes an immunosuppressive polyketide, which was shown to have a negative effect on in vitro murine T-cell proliferation [53]. This cluster was found up-regulated in V147(S)-ICZ and V147(S)-ISA.

### 2.7. Stress Response Pathways

**Figure 6:**
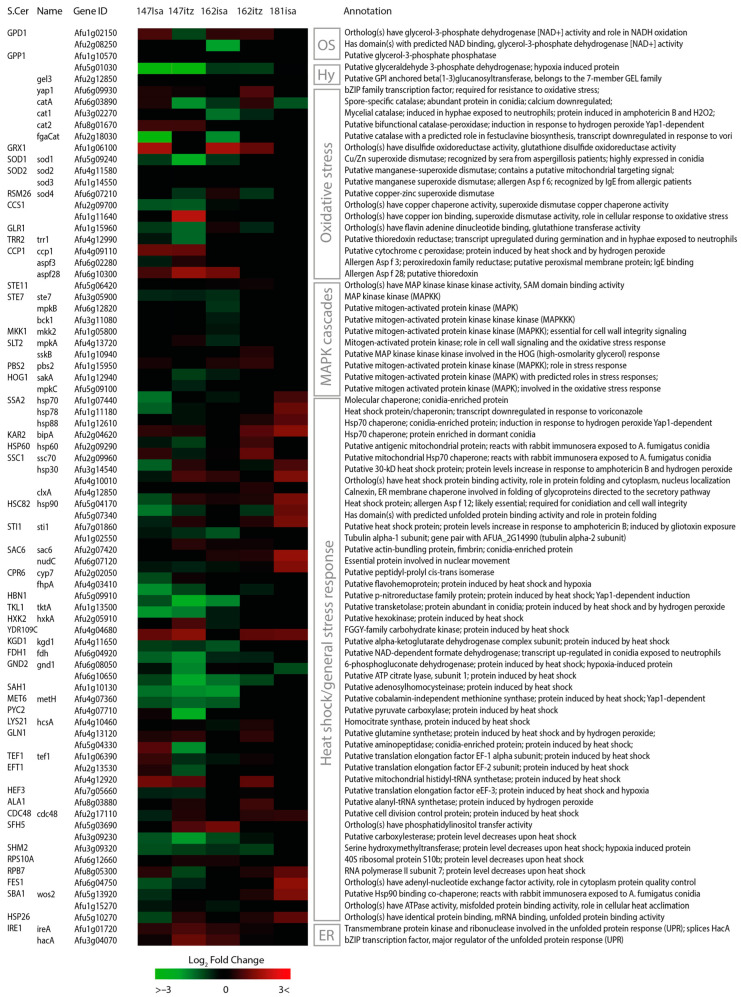
Stress response and signaling pathways. Heatmap of genes with a verified or predicted role in adaptation to several environmental stresses.

Fungi have several cellular mechanisms to cope with specific environmental stresses and adapt to unfavorable conditions. These mechanisms can be induced by specific conditions or are activated by a wider range of conditions [54]. Expression levels of genes associated with stress response pathways were assessed, such as several cellular stress responses, including high osmolarity adaptation, cell wall integrity maintenance, oxidative stress reduction, and heat shock tolerance [29,55]. We observed several stress-related genes to be affected by azole compounds (Figure 6).

The putative glycerol-3-phosphate dehydrogenase GfdA (Afu1g02150) is involved in the osmotic stress response and showed moderate up-regulation in V147(S)-ISA (Log2FC 0.98), V162(R)-ISA (Log2FC 0.70), and V162(R) ICZ (Log2FC 0.73). We found strong downregulation of a putative glyceraldehyde 3-phosphate dehydrogenase (Afu5g01030) in V147(S)-ISA, V147(S)-ICZ, V162(R)-ISA, and V162(R)-ICZ by log2FC −5.60, −5.59, −1.29, and −1.35, respectively. This gene traditionally functions in glycolysis, and its transcription is inhibited in the presence of an oxidant in *S. cerevisiae* [56]. *A. fumigatus* has two orthologous genes, *gpdA* (Afu5g01970) and *gpdB* (Afu8g02560), which are differentially regulated by log2FC −1.05 and log2FC 1.12 in V147(S)-ICZ in our dataset. 

The transcription factor AfYap1 is the main regulator of the oxidative stress response in A. fumigatus [57,58] and was moderately up-regulated in isolates V147(S)-ISA (0.51), V147(S)-ICZ (0.39) and V162(R)-ICZ (0.99). Several target genes of AfYap1 with antioxidant properties were up-regulated in isolates V147(S) and V162(R), which will be discussed below. The putative catalase Cat1 (Afu6g03890) was found down-regulated in isolates V147(S)-ICZ (log2FC−1.82), V162(R)-ISA, and in V181(R^PAN^)-ISA (log2FC−1.05). The putative catalase-peroxidase Cat2 (Afu8g01670) was found up-regulated only in isolates V147(S)-ISA and V147(S)-ICZ, with log2FC 0.80 in both treatments. Additionally, the mitochondrial cytochrome c peroxidase gene ccp1 was found up-regulated in V147(S)-ISA and V147(S)-ICZ by log2FC 1.27 and 1.23, respectively. Lastly, the mitochondrial peroxidase gene *prx1* (Afu4g08580) is also regulated by AfYap1 but was down-regulated in V147(S)-ISA, V147(S)-ICZ, and V162(R)-ISA by log2FC −0.86, −1.31, and −2.02, respectively. The putative glutaredoxin Grx1 (Afu1g06100) was found up-regulated in V147(S)-ISA, V162(R)-ISA, and V162(R)-ICZ, by log2FC 1.95, 1.94, and 1.21, respectively. Additionally, we found up-regulation in the thioredoxin allergen Aspf28 (Afu6g10300) in V147(S)-ISA, V147(S)-ICZ, and V162(R)-ISA by log2FC 0.9, 1.76, and 1.37, respectively. We did not find differential regulation of the superoxide dismutase (SOD) genes identified in A. fumigatus, except the putative SOD (Afu1g11640), which was found up-regulated only in isolate V147(S)-ICZ with log2FC 2.13.

### 2.8. The ER Stress Response

The unfolded protein response (UPR) is activated upon ER stress due to misfolded proteins, induces genes involved in lipid biosynthesis and ER-associated protein degradation (ERAD), and thereby prevents the formation of toxic protein aggregates [59]. Therefore, expression levels of genes associated with the ER stress response were assessed. The ER sensory protein IreA detects unfolded proteins and induces translation of the transcription factor HacA, which activates the expression of target genes with an unfolded protein response element (UPRE) [59,60]. *ireA* and *hacA* were found up-regulated in both treatments of isolates V147(S) and V162(R), with the most up-regulation found in isolate V147(S)-ICZ with log2FC 0.98 and 1.31, respectively (Figure 6). Furthermore, the UPR Hsp70 chaperone BiP/Kar2 that resides in the ER was found up-regulated in V162(R)-ICZ and V181(R^PAN^)-ISA, with log2FC 1.06 and 1.58, respectively. The unfolded protein response (UPR) protein OrmA (Afu4G13270) [60] was found up-regulated in isolate V147(S)-ICZ by log2FC 0.95. This protein is also involved in regulating sphingolipid biosynthesis, an important membrane lipid that associates closely with ergosterol in the membrane [61].

Additionally, the chaperone PdiA, which functions in ER-associated degradation of proteins (ERAD) (Afu2g06150), was found up-regulated by log2FC 1.14 in isolate V147(S)-ICZ. We found several genes present in the ubiquitin-proteasome pathway to be up-regulated. Ubiquitin conjugating enzymes (E2) UbcA (Afu5g07040) and UbcC (Afu5g09200) were found up-regulated in V147(S)-ICZ and V162(R)-ISA. UbcA was found up-regulated in V147(S)-ICZ and V162(R)-ISA by log2FC 1.12 and 1.09, respectively. UbcC was found up-regulated in V147(S)-ICZ by log2FC 1.29. The homolog of an E3 ligase specifically involved in ERAD in *S. cerevisiae,* Doa10 (Afu2g08650) [62], was found up-regulated in isolate V147(S)-ICZ and V162(R)-ISA with log2FC 2.79 and 1.33, respectively. Furthermore, we found up-regulation of two homologs of the cytosolic SCF ubiquitin E3 ligase complex subunit, CulA (Afu1g12960) and SkpA (Afugg06060), in V147(S) and V162(R) [63]. The fungal-specific F-box protein Fbx15 (Afu3g14150) is part of the SCF complex and is essential during stress adaptation [64]. We found an up-regulation of log2FC 0.91, 1.37, and 1.29 in V147(S)-ICZ, V147(S)-ISA, and V162(R)-ISA, respectively. Furthermore, the transcriptional repressor SsnF (Afu2g11840) was found down-regulated in V147(S)-ICZ by log2FC −1.07, which regulates the derepression of stress response genes and secondary metabolite genes [64]. 

Another important stress adaptation mechanism comprises the heat shock response, which is induced when environmental temperatures rise but is also activated upon stress conditions that interfere with protein integrity [65]. Heat shock proteins (HSPs) help fold proteins to their final state, aid in the stabilization of unfolded proteins, and prevent the accumulation of protein aggregates [66]. We observed the up-regulation of many essential proteins involved in the heat shock response and protein folding, including the highly conserved cytoplasmic chaperone Hsp70, the Hsp70 chaperone BiP/Kar2, molecular chaperone Hsp90, and Hsp90 co-chaperone Cdc37 (Figure 6). The Hsp70 chaperone BiP/Kar2 that resides in the ER was found up-regulated in V147(S)-ISA, V147(S)-ICZ, V162(R)-ICZ, and V181(R^PAN^)-ISA, as mentioned. We found the most up-regulation of heat-shock-response-related proteins in the pan-azole-resistant isolate V181(R^PAN^) after ISA treatment. Additionally, we found up-regulation of the mitochondrial Hsp60 chaperone in the V162(R)-ICZ isolate by log2FC 0.80. Lastly, the mitochondrial alpha-ketoglutarate dehydrogenase complex subunit Kgd1 (Afu4g11650) was found up-regulated in V147(S)-ISA, V147(S)-ICZ, V162(R)-ICZ, and V181(R^PAN^)-ISA by log2FC 1.23, 1.61, 1.17, and 1.21, respectively. This protein complex is induced by heat shock conditions, functions to form succinyl-CoA in the TCA cycle, and was found to function as an oxidative stress sensor in mammals [67,68,69]. Furthermore, it was found to bind specifically to respiratory complex I in mammals [70].

### 2.9. Gene Ontology (GO) Term Enrichment Analysis

In order to see if any biological processes were significantly altered that were not part of our genomic analysis based on current knowledge of virulence and resistance development, a GO term enrichment analysis was performed; see Table S7. All significant up- and down-regulated DEGs were analyzed separately for the ‘Biological process’, ‘Molecular function’, and ‘Cellular component’ categories. 

Many enriched categories in all treated isolates represent broad functional categories or are related to the process of ribosome synthesis and transcription itself, for instance, “GO:0005575 cellular_component, “GO:0002182 cytoplasmic translational elongation”, and “GO:0003735 structural constituent of ribosome”, which are the most enriched categories of isolate V147(S) for all up- and down-regulated genes after treatment with ICZ or ISA. Enriched GO terms that were found that described more specific cellular processes included “GO:0005739 mitochondrion”, “GO:0044550 secondary metabolite biosynthetic process”, “GO:0010106 cellular response to iron ion starvation”, “GO:0009986 cell surface”, “GO:0055085 transmembrane transport”, “GO:0051082 unfolded protein binding”, and “GO:0034605 cellular response to heat”. As no specific trends or overlapping enriched categories were seen between isolates, apart from the categories mentioned above that have been described in the results, we did not assess this data further.

## 3. Discussion

*A. fumigatus* has evolved molecular mechanisms to adapt its cellular physiology and overcome stressful environments. Our transcriptome study has provided insights into the transcriptional responses and molecular mechanisms that contribute to the survival of *A. fumigatus* hyphal cells during exposure to azole compounds. The most predominantly differentially regulated processes included sterol metabolism, siderophore-mediated iron acquisition, and secondary metabolite production. Several ABC and MFS transmembrane transporters were up-regulated, which implies increased efflux of cytotoxic compounds and possibly altered nutrient uptake. Furthermore, a variety of genes involved in several evolutionary conserved stress pathways was found up-regulated in varying degrees across tested *A. fumigatus* isolates.

Our exploratory analysis showed that the isolates tested show distinct background expression, as the sample factor appears to have a larger influence on sample distances than the treatment factor, which could be explained by differences in epigenetic regulation between isolates, as this is known to be a large factor in determining transcriptional variation [71]. Additionally, the basal differences in gene expression may also be due to the overexpression of *cyp51A* in the resistant isolates, as this may cause adaptive changes in the expression levels of other genes. Although we observed similarities in gene regulation between treated isolates, many DEGs appear to be specific for one sample and one treatment. This observation is partly explained by genetic variation for transcriptional plasticity, which describes genes that respond differently to changing environments depending on the isolate [72]. Although a general environmental stress response is observed in *S. cerevisiae* [73], this is not clearly observed in *Aspergillus* species to date [74]. In this study, the observed results were not validated by qPCR, as previous experiments by us and other groups have shown the validity of RNA-seq methods for *Aspergillus* spp. ([29,36,75,76,77,78,79,80,81]). In addition, the benchmark study conducted by Evereart et al., 2017 (Sci Rep) [82] showed a high concordance (~80%) between RNA-seq and qPCR data for various workflows and mapping tools. Of all non-concordant genes, the majority had a fold change of <2, which led to the observation that only 1.8% of the total gene set (13,045) in this paper showed a significantly different fold change when RNA-seq results were compared to RT-PCR results. In our dataset, we observed the most DEGs in V147(S)-ICZ, V147(S)-ISA, and V162(R)-ISA, suggesting that these isolates suffer the most stress and need to severely alter their metabolism. Overall, we found various similar differentially regulated processes in isolates V147(S) and V162(R) after azole treatments, including increased up-regulation in ergosterol biosynthesis genes as shown in previous research [83]. Isolates V147(S)-ISA, V147(S)-ICZ, and V162(R)-ISA showed up-regulation of the majority of genes involved in ergosterol biosynthesis, whereas itraconazole-resistant isolate V162(R)-ICZ showed only strong up-regulation for the *erg6* gene (Afu4g03630). Our previous study on the immediate transcriptomic response of *A. fumigatus* to itraconazole also showed up-regulation of *erg6* within 30 min of ICZ addition to the medium, which persisted until 240 min after azole addition [36]. The Erg6 enzyme functions directly upstream of Cyp51A in the sterol biosynthesis pathway in *A. fumigatus*, where it methylates lanosterol at C24 to form eburicol [13]. It remains unclear specifically why *erg6* is strongly up-regulated upon azole perturbation, although this was also observed in previous studies [36,83,84]. This enzyme is a potential antifungal target, as Erg6 catalyzes a reaction that is not present in the cholesterol biosynthesis pathway [85].

Previous research showed that repression of *erg11* in a conditional mutant of *Neurospora crassa* results in a similar up-regulation of *erg6* by log2FC 5.1, as well as up-regulation of other *erg* genes in this pathway. The authors show that the transcriptional response of ergosterol biosynthesis genes is not a direct result of azole drug presence or ergosterol depletion but rather the consequence of accumulated sterol intermediates in the cell [83]. However, direct evidence for this theory still has to be provided for *A. fumigatus*. Interestingly, pan-azole-resistant strain V181(R^PAN^) did not show any differential regulation in ergosterol biosynthesis, which could be explained by the lack of intermediate sterols accumulating in the cell, although further research is required to support this notion. In this isolate, the TR46^3^ in the promotor region of the *cyp51A* gene is responsible for a strong increase in the Cyp51A enzyme, which possibly ensures a continuous ergosterol biosynthesis despite a high concentration of azoles present in the cell. In addition, the V181(R^PAN^) isolate showed differential expression of several hundred genes when treated with isavuconazole but not when treated with itraconazole, even though its genotype confers resistance to both azoles. An explanation for this might be that the growth observed in a microtiter plate from which the IC50 concentrations are derived is not an accurate predictor for transcriptome changes taking place in the mycelium, other than the absence of differential regulation in the ergosterol biosynthesis pathway.

The siderophore biosynthesis pathway is involved in iron acquisition when the intracellular iron concentration is low and shares mevalonate as an important substrate with the ergosterol biosynthesis pathway [44]. Adaptation to limited iron availability is essential to maintain fungal virulence [42]. During infections, the innate immune system is able to restrict iron availability to counteract infections, through iron-chelating proteins secreted by macrophages and neutrophils, underlining the importance of these mechanisms in *A. fumigatus* [86].

We observed downregulation of the majority of *sid* genes and siderophore importer MirB in isolates V147(S) and V162(R) during both treatments, implying a metabolic shift for mevalonate as it is preferentially converted to ergosterol upon ergosterol biosynthesis inhibition by azoles. This observation suggests that mevalonate is a key metabolite in susceptible and resistant isolates during azole therapy. Furthermore, these data show that *A. fumigatus* does not suffer from iron depletion under these conditions, as up-regulation of genes involved in siderophore biosynthesis and siderophore-mediated iron uptake would be expected.

The involvement of transmembrane transporters in antifungal resistance has been elucidated over the past decades. *A. fumigatus* has various drug transporters involved in azole resistance, including AbcA (Afu2g15130) and Cdr1B (Afu1g14330) [87]. Our findings included significant up-regulation of these transporters during both ICZ and ISA treatment in all isolates. A recent study on heterologously expressed *A. fumigatus* transporters in a ΔPDR5 *S. cervisiae* isolate showed that expression of *abcA* (Afu2g15130) only resulted in a slight increase in the posaconazole MIC [88], a finding that is in contrast with an earlier study that showed voriconazole sensitization of an *abcA* knockout strain [87]. A recent study created tetracycline-inducible *A. fumigatus* isolates of seven ABC transporters and found increased growth on solid agar supplemented with voriconazole in the strains overexpressing *abc1* (Afu3g01400), *abc3* (Afu6g08020), *abc4* (Afu4g14130), and *abc5* (Afu6g03080) [28]. We found up-regulation of *abc1* in V147(S)-ISA and V162(R)-ISA but downregulation in V147(S)-ICZ, indicating that this gene is not up-regulated by ICZ-induced stress. Esquivel et al. showed that this *abc1* transporter (dubbed *abcH* in their study) had a narrow substrate range and even had a negative impact on the ICZ MIC [88], which could explain the difference in regulation after ICZ or ISA treatment seen in our dataset. In the same study, the expression of *abcF* (Afu5g00790) or *abcC*/*cdr1B* (Afu1g14330) was found to elevate MICs of all azoles tested, but whereas *abcC* was found up-regulated in all our isolates, *abcF* was found down-regulated in the majority of our isolates. This shows that efflux transporters that can increase the azole MICs of an isolate when artificially up-regulated in the laboratory are not necessarily up-regulated naturally by *A. fumigatus* upon azole addition to the medium.

We also found up-regulation of MFS transporter *mdr1* (Afu5g06070) in all isolates during ICZ and ISA treatment, although a recent study showed that a conditional Mdr1_TetOn_ strain had no influence on voriconazole susceptibility compared to the wildtype [28]. This implies that *mdr1* is activated as the result of a general stress response but is not essential for azole resistance. Interestingly, we found the highest up-regulation of *abcE* (Afu7g00480) with log2FC 4.32 in pan-azole-resistant isolate V181(R^PAN^) during ISA treatment, whereas this gene was only moderately up-regulated in all other isolates. Previously, *abcE* was found up-regulated in response to voriconazole in the medium [34]. In *A. niger*, a syntenic homolog of this gene is present (An12g03150), which is induced after dithiothreitol (DTT) treatment, a compound that induces the ER stress response [89]. This could indicate that *abcE* is up-regulated due to an activated UPR pathway in isolate V181(R^PAN^).

Overall, we found differential regulation of many membrane transporters with an unknown function upon azole addition. Recently, it was observed that CDR drug transporters in *C. albicans* were transcriptionally activated upon accumulation of toxic sterol intermediates, an indirect effect resulting from azole treatment [83]. This demonstrates that transporters that are up-regulated upon azole treatment are not necessarily involved in azole efflux directly but are involved in the efflux of accumulated toxic sterol intermediates or are activated by general stress responses. 

These results highlight that several transmembrane transporters that were shown to increase the MIC for azole compounds are transcriptionally activated and that general stress response mechanisms can up-regulate transporters that do not provide efflux of azole compounds or directly improve growth in the proximity of azole compounds. Secondary metabolites can provide protection against environmental stresses and microorganisms, and the production of these small bioactive molecules is known to increase virulence in *A. fumigatus* [47,50,90]. Secondary metabolite production upon azole addition in the medium has not been reported to date. We found up-regulation of the fumisoquin biosynthetic cluster (BCG18) in isolates V147(S)-ISA, V147(S)-ICZ, and V162(R)-ISA, indicating that these compounds can be produced in susceptible isolates and resistant isolates upon azole treatment. Overexpression of the transcriptional regulator *fsqA* did not increase virulence in a neutropenic murine model [91]. The *has* cluster was up-regulated in V147(S)-ICZ and V162(R)-ISA, indicating that the cell increases iron-binding hexadehydroastechrome production, perhaps to counter a down-regulated siderophore production, which limits iron acquisition. The has cluster was also found to be up-regulated after iron starvation and H_2_O_2_-induced oxidative stress [92]. For reasons unknown, up-regulation of the pyripyropene A biosynthetic cluster was only found in V147(S)-ICZ, in contrast to isolates V147(S)-ISA and V162(R)-ISA where this cluster was found to be down-regulated. Up-regulation of neosartoricin/fumicycline A biosynthetic cluster was solely found in isolate V147(S), indicating that the production of this compound after azole addition is specific for this isolate. Interestingly, we found up-regulation of several genes present in the gliotoxin biosynthetic cluster in V147(S)-ISA, V147(S)-ICZ, and V162(R)-ICZ, although this up-regulation could not be rendered as significant in many instances due to a low transcript count (Table S5). Previous research has shown that *A. fumigatus* produces more gliotoxin when grown in a biofilm than when grown planktonic in a liquid medium, which could explain why we observed low quantities of gliotoxin-related gene transcripts [93]. We show that azole treatment of susceptible and resistant isolates can result in the up-regulation of several secondary metabolite-encoding biosynthetic clusters.

Several antifungal compounds have been reported to induce the formation of reactive oxygen species (ROS) in the cell as a secondary mechanism of antifungal activity. Intracellular ROS causes lipid peroxidation, which ultimately threatens the integrity of membranes [94]. Among the azole class antifungals, micafungin was shown to induce ROS species in *C. albicans* biofilms [95], and itraconazole was shown to induce ROS in *A. fumigatus*, although to a lesser extent than terbinafine and amphotericin B [96]. In our dataset, we observed up-regulation of transcription factor Yap1 (Afu6g09930) in isolate V162(R)-ICZ, which translocates from the cytoplasm to the nucleus upon oxidative stress [26]. We also observe up-regulation of catalases CatA (Afu6g03890) in isolate V162(R)-ICZ; Cat2 (Afu8g01670) in isolates V147(S)-ISA and V147(S)-ICZ; uncharacterized glutaredoxin ‘GRX1’ (Afu1g06100) in the majority of isolates; glutaredoxin *grxD* (Afu2g14960) in isolates V147(S)-ISA, V147(S)-ICZ, and V162(R)-ICZ; the mitochondrial cytochrome C peroxidase (Afu4g09110) in isolates V147(S)-ISA and V147(S)-ICZ; an uncharacterized superoxide dismutase (Afu1g11640); and the putative thioredoxin Aspf28 (Afu6g10300) in V147(S)-ISA, V147(S)-ICZ, and V162(R)-ISA. This indicates that these isolates experience oxidative stress upon azole addition to the medium. This observation corresponds with the hypothesis that ROS are produced by membrane-perturbating compounds that cause the destabilization of membranes and the mitochondrial complex I [96]. However, the up-regulation of these genes was found to be variable between isolates and treatments. In *A. fumigatus*, the SOD genes have been observed to mainly counteract oxidants, which naturally arise in the cell due to metabolism and respiration, rather than detoxify ROS as part of a stress response pathway [97]. Instead, thioredoxins and glutaredoxins could be playing a larger role in detoxifying stress-induced ROS. We found up-regulation of an uncharacterized homolog of GRX1 (Afu1g06100) and the essential glutaredoxin *grxD* (Afu2g14960); the latter was found to be up-regulated under iron starvation conditions when it functions to activate HapX. The overexpression of this gene was not found to influence other genes under iron-replete conditions [92].

We found differential regulation of genes involved in the ER stress response and the unfolded protein response (UPR), which is activated upon ER stress. Upon ER stress, the expression of constitutively active heat shock proteins is up-regulated, which are essential in facilitating protein folding under normal conditions and protecting the cell by stabilizing unfolded proteins [69]. 

We found up-regulation of *ireA* (Afu1g01720) and *hacA* (Afu3G04070) in isolates V147(S) and V162(R) during both treatments. Furthermore, we found up-regulation of UPR protein *ormA* (Afu4g13270) in isolate V147(S)-ICZ. It was shown that the overexpression of *ormA* in *A. fumigatus* leads to reduced ICZ susceptibility [61].

The ER-resident HSP70 chaperone BiP/KAR2 is involved in protein folding, protein translocation across the ER membrane, and ER-associated degradation and was found up-regulated in the majority of isolates. In the pathogenic yeast *Cryptococcus neoformans*, a transcriptomics approach uncovered that azole treatment is sensed as ER stress, which activates Ire1 and results in the induction of KAR2 by the UPR pathway [98]. Indeed, induction of KAR2 was found to be essential to dispose of misfolded proteins, which alleviates ER stress [99]. Another key stress response protein Hsp90 (Afu5g04170) was found up-regulated, which is mainly involved in the final maturation of proteins and the assembly of complex macromolecular structures [66]. Furthermore, Hsp90 co-chaperone Cdc37 (Afu4g10010) and Hsp70/Hsp90 co-chaperone Sti1 (Afu7g01860) were found up-regulated, which are involved in the stabilization of kinases and protein aggregates and suppression of proteotoxicity from aggregated proteins, respectively [100]. In *T. rubrum*, *hsp90* deletion was found to have a profound effect on itraconazole tolerance [101]. Furthermore, DSC complex subunit ubiquitin ligase *dscA* (Afu1g12080) present in the Golgi apparatus was also found up-regulated in isolates V147(S) and V162(R) during both treatments. This complex is critical for the activation of SrbA by proteolytic cleavage and thus for the cellular response to azole drugs [102]. Lastly, the highly conserved ERAD-associated E3 ubiquitin ligase Doa10 was found up-regulated in isolates V147(S) and V162(R) during both treatments, which facilitates the degradation of Erg1 after lanosterol accumulation in order to prevent further accumulation of disruptive sterol intermediates in the cell [103].

## 4. In Summary

It appears that the transcriptome response is in part determined by the genomic background of the isolate. Transcriptional plasticity was shown to be more variable in multi-copy and non-essential genes [72], which might further influence the transcriptional response we observed. This underlines the importance of the characterization of genes as it enables us to better understand physical adaptation through transcriptional regulation. These data show that the pan-azole-resistant isolate with the highest MICs for azoles shows the least amount of DEGs when confronted with azole compounds, and our susceptible isolate V147(S) with the lowest MICs for azoles displayed the highest amount of DEGs, with ICZ-resistant isolate V162(R) being in the middle.

We found that many HSPs were up-regulated in pan-azole-resistant isolate V181(R^PAN^) harboring a TR46^3^, originally isolated from the environment [30]. It is possible that hyperactivation of *cyp51A* transcription under azole growth pressure causes increased protein misfolding and ER stress; hence, HSPs are activated to alleviate ER stress [104]. These observations fall in line with the strong up-regulation that was found in this isolate for *abcE*, a transporter that was shown to be up-regulated in *A. niger* after DTT-induced ER stress [89]. Additionally, it could reflect adaptation to the environment that this strain has been isolated in, a compost heap that can reach temperatures of up to 70 °C [30]. Interestingly, this mechanism has been observed before in thermotolerant yeasts, that global transcription changes as a ‘preventive response’ contributed to growth at elevated temperatures [105]. Interestingly, we found no differential regulation of *cyp51A* or any other genes involved in ergosterol biosynthesis in V181(R^PAN^) after azole addition to the medium, underlining that there might be no disruption in ergosterol biosynthesis in this strain when confronted with isavuconazole, as compared to the untreated isolate. However, as the base-level expression of hundreds of genes including *cyp51A* in isolate V181(R^PAN^) are differentially expressed when compared to isolate V147(S) as shown in Table S6, it is plausible that this isolate is already equipped to encounter azole compounds in the environment, which would remove the need for *A. fumigatus* to further alter its transcription of ergosterol biosynthesis genes.

Considering azole-resistant *A. fumigatus* isolates, many factors that determine the resistance phenotype remain unknown. We found diverse transcriptomes after treating three *A. fumigatus* isolates with a distinct genetic background with azole antifungal compounds. Although several cellular processes were subjected to similar regulation, including ergosterol biosynthesis up-regulation and siderophore biosynthesis downregulation, many isolates have a transcriptome unique for that isolate. 

Therefore, it is important that the transcriptional response of more clinical and environmental isolates is measured. Additional RNA-seq data could provide information on the diversity of the transcriptional response when *A. fumigatus* is confronted with azole compounds to uncover possible core gene sets that are transcriptionally adjusted in isolates with similar genetic backgrounds or comparable azole susceptibilities.

## 5. Conclusions

Several cellular mechanisms are involved in the fungal stress response against azole compounds, and determining their contribution to the resistant phenotype is valuable in being able to recognize more targets for antifungal drug development and optimize azole therapy.

Monitoring transcriptome changes could help us understand which changes in gene expression have a strong influence on the phenotype and which transcriptional changes allow for better adaptation and survival of the fungus in the presence of azoles.

## 6. Material and Methods

### 6.1. Isolates, Media, and Culture Conditions

Three isolates with variable susceptibilities for itraconazole (Sigma-Aldrich, Zwijndrecht, The Netherlands) and isavuconazole (Sigma-Aldrich, Zwijndrecht, The Netherlands) were selected, with isolates V147(S) and V162(R) of clinical origin and pan-azole-resistant isolate V181(R^PAN^) of environmental origin. All isolates are listed in Table 1. Isolates were cultured on either Sabouraud Dextrose Agar (SDA) (Oxoid, Landsmeer, The Netherlands) (1% peptone, 4% glucose, 1.5% agar, pH 5.6) or Aspergillus Minimal Medium (AMM) as described in the supplemental material of [106] containing per liter: 10 g glucose, 5.95 g NaNO_3_, 0.522 g KCl, 1.5 g KH_2_PO_4_, 50 mg MgSO_4_·7H_2_O, and 1 mL trace elements. Trace elements contained (per 200 mL): 10 g EDTA, 4.4 g ZnSO_4_·7H_2_O, 1.01 g MnCl_2_·4H2O, 0.315 g CuSO_4_·5H_2_O, 0.22 g (NH4)6Mo_7_O_24_·4H_2_O, 1.0 g Fe(II)- SO_4_·7H_2_O, and 2.2 g H_3_BO_3_ [107]. All compounds were produced by Merck (Darmstadt, Germany). Isolates were stored in 20% glycerol at −80 °C and subcultured on SDA at 37 °C for 4–5 days. Conidia were harvested with a wet cotton swab and resuspended in Milli-Q containing 0.1% Tween 20. Approximately 2.6 × 10^5^ CFU/mL of conidia were used to inoculate 5 mL of AMM in 15 mL tubes, containing IC_50_ concentrations of ICZ or ISA or the same volume of DMSO as a control. Conidia were inoculated in duplo for each condition. After 48 h of growth at 37 °C while rotating, mycelial bulbs were harvested through Miracloth (Merck) and directly frozen in liquid nitrogen.

### 6.2. Dose–Response Curves

For determining the ICZ and ISA concentrations where growth is estimated to be 50% (IC50), dose–response curves were made by growing each strain (n = 5) in a 96-well plate containing AMM with itraconazole concentrations ranging from 0.016 μg/mL to 16 μg/mL. Plates were kept at 37 °C in a microtiter plate reader (Anthos Labtec Instruments GmbH, Salzburg, Austria) for 48 h, and optical density (OD) was measured every hour at 405 nm. After subtraction of the ODs of the empty wells, the percentage of growth from the ODs of the inoculated wells for each well was correlated with the relative OD estimated by using the following equation: (OD405 of wells that contained itraconazole/OD405 of the itraconazole-free well) × 100. The relationship was determined by anon-linear regression analysis and the Hill equation with a variable slope fitted to the data. Goodness of fit was checked by the R^2^ values. All analyses were performed by using GraphPad Prism, version 6.0, for Windows (GraphPad Software, San Diego, CA, USA). For isolate V181(R^PAN^), a treatment concentration of 16 µg/mL ISA was used due to the 0 DEGs that were found after 8 µg/mL ICZ.

### 6.3. RNA-Sequencing

After harvesting, mycelia were lysed by bead-bashing using a MagNa Lyser instrument three times for 30 s at 7000 rpm (Roche, Basel, Switzerland). RNA was extracted using TRIzol reagent (Invitrogen, Breda, the Netherlands). Pellets were resuspended in 100 μL of DEPC-treated water. RNA integrity was verified through agarose gel electrophoresis. Total RNA was stained with Midori Green (Nippon Genetics, Dueren, Germany) and visualized with UV light. The purity of all samples (A260/A280 ≥ 1.5 and A260/A230 ≥ 2.0) was determined using a NanoDrop 1000 (Thermo Scientific, MA, USA). Pooled cDNA libraries were constructed with the TruSeq RNA Sample Prep Kit v2 (Illumina, San Diego, CA, USA) according to manufacturer instructions. The quality of the libraries was verified with an Agilent 4200 Tapestation System (Agilent Technologies). For our ICZ-treated isolates, paired-end reads of 2 × 75 bp were generated in High Output mode on a NextSeq 500 sequencer (Illumina, San Diego, CA, USA) at the Genome Diagnostics and the Core Genome Analysis Laboratory of Radboudumc. For our ISA-treated isolates, paired-end reads of 2 × 100 bp were generated on a HiSeq 4000 (Illumina, San Diego, CA, USA) at BGI Genomics (Copenhagen, Denmark). 

### 6.4. Identification of Differentially Expressed Genes

Reads were aligned with STAR v2.5.3A [108] against the reference genome sequence of *A. fumigatus* Af293 Ensemble CADRE 30 downloaded from Ensembl Fungi. A batch correction was additionally performed by using DESeq2 in programming language R by Bioconductor [109]. Differentially expressed genes (DEGs) were identified by DESeq2 (BaseMean ≥ 36, *p*-value < 0.05, adjusted *p*-value < 0.1, log2FC > 0.7 or <−0.7), and results were corrected for multiple testing by the Benjamini–Hochberg principle. A batch correction was additionally performed by DESeq2 to correct for sequencing artifacts created by the difference in batches. Treatment with ISA and ICZ was performed in two separate batches; hence, there are four replicates total of the untreated (DMSO) condition per isolate. The untreated isolates per strain were grouped together for the DESeq2 analysis to increase statistical power. Functional categories for filamentous fungi, including *A. fumigatus*, remain poorly defined. Therefore, a more directional approach was used by assessing pathways associated with stress and virulence based on current knowledge. 

GO term analysis was performed in FungiFun V2.2.8 [110]. Fisher’s exact test was performed with a Benjamini–Hochberg procedure for multiple-testing error correction for enriched categories in the ‘Biological process’, ‘Cellular component’, and ‘Molecular function’ ontologies. Statistical analysis, heatmaps, and volcano plots were generated in Rstudio version 1.2.1335 for Windows 10. For generating heatmaps, gplots v3.0.1 was used. For generating volcano plots, ggplot2 V2.2.1 was used. Exported images were assembled with Adobe Illustrator CC 2015. Venn diagrams were created with the Venn tool by (http://bioinformatics.psb.ugent.be/webtools/Venn, accessed on 22 March 2022). Graphs of dose–response curve analyses were generated by using GraphPad Prism, version 6.0, for Windows (GraphPad Software, San Diego, CA, USA).

### 6.5. Microsatellite Genotyping

Short Tandem Repeat (STR) analysis was performed on the isolates used in this study.

Multiplex PCR was performed for *A. fumigatus* STR loci; three trinucleotide (STRAf3A-C) and three tetranucleotide loci (STRAf4A-C) were amplified using corresponding primers containing the dye carboxyfluorescein (FAM), hexachlorofluorescein (HEX), or tetrachlorofluorescein (TET) at the 5′ end [111]. Cleaned PCR products were analyzed together with a GeneScan500 LIZ size standard (Applied Biosystems, Foster City, CA, USA), and fluorescence was detected on a Genetic Analyzer (Applied Biosystems, Foster City, CA, USA). The number of repeats was determined using the Peak Scanner Software v1.0 (Applied Biosystems, Foster City, CA, USA).

## Figures and Tables

**Table 1:** Isolates used in this study.

	MIC												TREATED WITH	
ISOLATE	ICZ	VOR	POS	ISA	Cyp51A Genotype	STR3A	STR3B	STR3C	STR4A	STR4B	STR4C	Reference	ICZ (IC50)	ISA (IC50)
V147(S)	0.5	1	0.25	1	WT	23	6	14	5	4	4	This study	0.15 µg/mL	0.10 µg/mL
V162(R)	>16	4	0.25	8	TR34/L98H	52	9	11	7	6	11	This study	8 µg/mL	1.98 µg/mL
V181(R)	>16	>16	1	>16	TR46^3^/Y121F/M172I/T289A/G448S	26	8	12	6	7	7	[30]	8 µg/mL	16 µg/mL

**Table 2:** List of the 50 DEGs with the highest and lowest log2FC for each isolate.

		V147(S)-ICZ	
		Up-regulated	
2logFC	gene	Description	Name
10,18	Afu2g17930	Integral membrane protein	N/A
9,15	Afu3g12910	O-methyltransferase GliM-like; putative	hasC
8,26	Afu8g06070	Putative uncharacterized protein	N/A
7,65	Afu4g02720	GPI anchored glycosyl hydrolase; putative	N/A
7,29	Afu3g12920	Nonribosomal peptide synthetase 5	nrps5
7,26	Afu2g10130	Adhesin; putative	N/A
7,25	Afu3g12900	MFS transporter; putative	hasB
7,19	Afu3g12930	7-dimethylallyltryptophan synthase	hasE
7,17	Afu3g12960	Cytochrome P450 monooxigenase GliC-like; putative	hasH
6,97	Afu7g04550	Serine/threonine protein kinase; putative	N/A
6,82	Afu6g11850	Putative uncharacterized protein	N/A
6,62	Afu7g04570	Na/K ATPase alpha 1 subunit; putative	N/A
6,45	Afu3g01500	Integral membrane protein	N/A
6,41	Afu4g01260	Mitochondrial chaperone ATPase (Bcs1); putative	bcs1
6,40	Afu3g01960	Putative uncharacterized protein	N/A
6,32	Afu8g00620	Dimethylallyl tryptophan synthase; putative	cdpNPT
6,24	Afu6g14140	RTA1 domain protein; putative	N/A
5,81	Afu4g01290	Glycosyl hydrolase family 75 chitosanase; hydrolyzes beta-1,4-glycosidic linkage of chitosan; repressed by exposure to artemisinin	csnB
5,72	Afu5g02330	Ribonuclease mitogillin	aspf1
5,72	Afu2g17810	RTA1 domain protein; putative	N/A
5,55	Afu3g03470	Putative uncharacterized protein	N/A
5,52	Afu7g04560	Putative uncharacterized protein	N/A
5,47	Afu2g16540	C2H2 finger domain protein; putative	N/A
5,42	Afu1g01175	Putative uncharacterized protein	N/A
5,25	Afu4g14030	Putative uncharacterized protein	N/A
		Down-regulated	
-3,67	Afu8g05940	Putative uncharacterized protein	N/A
-3,71	Afu3g02800	Lipase/esterase; putative	N/A
-3,72	Afu6g04360	ABC drug exporter AtrF	atrF
-3,74	Afu2g00460	Short-chain dehydrogenase/reductase family protein; putative	N/A
-3,78	Afu4g01560	MFS myo-inositol transporter; putative	N/A
-3,79	Afu2g00550	Putative uncharacterized protein	N/A
-3,81	Afu6g07820	Integral membrane protein; putative	N/A
-3,81	Afu3g14940	Elastase inhibitor AFUEI	aeiA
-3,87	Afu1g03352	Alpha-1;3-glucanase/mutanase; putative	N/A
-3,89	Afu6g00730	Putative uncharacterized protein	N/A
-4,03	Afu4g13570	Thiol methyltransferase; putative	N/A
-4,09	Afu1g17180	Pyridine nucleotide-disulphide oxidoreductase AMID-like; putative	N/A
-4,31	Afu7g00700	Aldo-keto reductase (AKR13); puatative	akr13
-4,32	Afu2g01590	Non-classical export protein Nce102; putative	nce102
-4,64	Afu6g03350	GNAT family N-acetyltransferase; putative	N/A
-4,67	Afu7g01040	Cytidine deaminase; putative	N/A
-4,75	Afu4g00450	Putative uncharacterized protein	N/A
-4,86	Afu3g13620	Cupin domain protein	N/A
-5,15	Afu8g06760	MFS transporter; putative	N/A
-5,15	Afu8g01290	Putative uncharacterized protein	N/A
-5,25	Afu1g16440	ABC ATPase; putative	N/A
-5,41	Afu2g05290	Isoflavone reductase family protein	N/A
-5,59	Afu5g01030	Glyceraldehyde-3-phosphate dehydrogenase	N/A
-6,32	Afu8g01660	Putative uncharacterized protein	N/A
-6,43	Afu6g03190	Putative uncharacterized protein	N/A
		V147(S)-ISA	
		Up-regulated	
2logFC	gene	Description	
5,74	Afu1g01175	Putative uncharacterized protein	N/A
5,36	Afu2g17930	Integral membrane protein	N/A
5,20	Afu7g00160	Polyketide synthase; putative	nscA
4,99	Afu7g00120	Metallo-beta-lactamase domain protein	nscB
4,80	Afu7g00170	Dimethylallyl tryptophan synthase GliD1	nscD
4,54	Afu7g00180	NAD dependent epimerase/dehydratase; putative	nscE
4,46	Afu7g00200	Putative uncharacterized protein	N/A
4,36	Afu4g01260	Mitochondrial chaperone ATPase (Bcs1); putative	bcs1
4,16	Afu3g01960	Putative uncharacterized protein	N/A
4,13	Afu6g14140	RTA1 domain protein; putative	N/A
4,06	Afu8g00620	Dimethylallyl tryptophan synthase; putative	cdpNPT
4,01	Afu8g06640	UbiE/COQ5 methyltransferase; putative	N/A
3,98	Afu4g01290	Glycosyl hydrolase family 75 chitosanase; hydrolyzes beta-1,4-glycosidic linkage of chitosan; repressed by exposure to artemisinin	csnB
3,95	Afu6g03680	Putative uncharacterized protein	N/A
3,93	Afu7g04550	Serine/threonine protein kinase; putative	N/A
3,80	Afu5g01230	RTA1 domain protein; putative	N/A
3,71	Afu7g04570	Na/K ATPase alpha 1 subunit; putative	N/A
3,63	Afu7g04560	Putative uncharacterized protein	N/A
3,61	Afu3g01500	Integral membrane protein	N/A
3,56	Afu8g02070	Glycosyl transferase; putative	N/A
3,46	Afu4g02720	GPI anchored glycosyl hydrolase; putative	N/A
3,38	Afu4g09550	Putative uncharacterized protein	N/A
3,36	Afu1g12420	Putative uncharacterized protein	N/A
3,33	Afu4g03630	Sterol 24-c-methyltransferase; putative	erg6
3,25	Afu3g10370	Putative uncharacterized protein	N/A
		Down-regulated	
-2,60	Afu2g00570	GNAT family acetyltransferase; putative	N/A
-2,61	Afu2g17850	3-beta hydroxysteroid dehydrogenase/isomerase; putative	N/A
-2,71	Afu1g17270	FRE family ferric-chelate reductase; putative	fre2
-2,73	Afu5g11250	Polyglutamate biosynthesis protein; putative	N/A
-2,74	Afu6g00750	Pyruvate decarboxylase; putative	pdcB
-2,75	Afu4g12740	tRNA (Adenine-N(1)-)-methyltransferase	N/A
-2,77	Afu6g03350	GNAT family N-acetyltransferase; putative	N/A
-2,77	Afu3g03650	GNAT family acetyltransferase; putative	sidG
-2,79	Afu3g03420	Nonribosomal peptide synthetase 4	sidD
-2,80	Afu5g13970	WD domain; G-beta repeat protein	N/A
-2,83	Afu6g00760	Glutathione S-transferase; putative	N/A
-2,89	Afu3g15050	Flavin-binding monooxygenase; putative	N/A
-2,91	Afu1g17170	TfdA family taurine dioxygenase; putative	N/A
-2,94	Afu3g03660	Siderophore esterase IroE-like; putative	estB
-2,98	Afu3g15055	Putative uncharacterized protein	N/A
-3,03	Afu4g00450	Putative uncharacterized protein	N/A
-3,26	Afu2g00580	Putative uncharacterized protein	N/A
-3,28	Afu2g18030	Catalase	fgaCat
-3,37	Afu1g03352	Alpha-1;3-glucanase/mutanase; putative	N/A
-3,39	Afu3g14940	Elastase inhibitor AFUEI	aeiA
-3,89	Afu3g03640	MFS siderochrome iron transporter MirB	mirB
-3,96	Afu3g15010	Putative uncharacterized protein	N/A
-4,39	Afu6g03190	Putative uncharacterized protein	N/A
-4,87	Afu3g03010	Phosphate-repressible Na+/phosphate cotransporter Pho89; putative	Pho89
-5,60	Afu5g01030	Glyceraldehyde-3-phosphate dehydrogenase	N/A
		V162(R)-ICZ	
		Up-Regulated	
2logFC	gene	Description	
3,89	Afu4g03630	Sterol 24-c-methyltransferase; putative	erg6
3,43	Afu7g04560	Putative uncharacterized protein	N/A
3,24	Afu2g15130	ABC multidrug transporter; putative	abcA
2,86	Afu2g08680	Putative uncharacterized protein	N/A
2,85	Afu6g14140	RTA1 domain protein; putative	N/A
2,75	Afu3g11880	Putative uncharacterized protein	N/A
2,48	Afu2g15140	MFS drug transporter; putative	N/A
2,29	Afu8g06640	UbiE/COQ5 methyltransferase; putative	N/A
2,26	Afu2g12820	Polyketide synthase; putative	N/A
2,25	Afu1g17470	High affinity nitrate transporter NrtB	nrtB
2,18	Afu1g06480	Putative uncharacterized protein	N/A
2,18	Afu1g03270	Putative uncharacterized protein	N/A
2,15	Afu3g00710	Allergen Asp F4-like; putative	N/A
2,13	Afu6g09960	Putative uncharacterized protein	N/A
2,12	Afu7g04880	Sterol glucosyltransferase; putative	N/A
2,03	Afu6g11720	Putative uncharacterized protein	N/A
1,98	Afu7g00910	OPT peptide transporter Mtd1; putative	optH
1,97	Afu1g06470	Neutral/alkaline nonlysosomal ceramidase; putative	N/A
1,94	Afu1g11360	Aldehyde reductase II	N/A
1,93	Afu2g00240	Putative uncharacterized protein	N/A
1,91	Afu3g09240	CAIB/BAIF family enzyme	N/A
1,89	Afu2g00410	Amidase family protein	N/A
1,88	Afu4g01410	Putative uncharacterized protein	N/A
1,86	Afu3g07860	Glycosyl transferase; putative	gtb3
1,85	Afu8g01010	Thermophilic desulfurizing enzyme family protein	N/A
		Down-Regulated	
-1,73	Afu5g00580	Putative uncharacterized protein	N/A
-1,74	Afu6g03190	Putative uncharacterized protein	N/A
-1,76	Afu7g06920	NmrA family transcriptional regulator; putative	N/A
-1,76	Afu3g12255	Putative uncharacterized protein	N/A
-1,77	Afu1g10380	Nonribosomal peptide synthetase 1	nrps1
-1,86	Afu8g05520	Aldehyde dehydrogenase family protein	N/A
-1,87	Afu7g06350	Sodium/phosphate symporter; putative	phoE
-1,88	Afu4g01350	Putative uncharacterized protein	gprK
-1,95	Afu4g00440	Short chain dehydrogenase; putative	N/A
-2,06	Afu2g17900	Putative uncharacterized protein	N/A
-2,07	Afu3g03640	MFS siderochrome iron transporter MirB	MirB
-2,07	Afu7g06150	Endoglucanase; putative	N/A
-2,14	Afu5g01620	Extracellular proline-rich protein	N/A
-2,14	Afu4g01360	MFS transporter of unkown specificity	N/A
-2,16	Afu1g10390	ABC multidrug transporter; putative	abcB
-2,20	Afu7g04920	Putative uncharacterized protein	N/A
-2,22	Afu2g00580	Putative uncharacterized protein	N/A
-2,25	Afu1g04130	FG-GAP repeat protein; putative	N/A
-2,32	Afu5g14740	Fucose-specific lectin FleA	fleA
-2,33	Afu1g03352	Alpha-1;3-glucanase/mutanase; putative	N/A
-2,53	Afu6g03560	IgE-binding protein; putative	N/A
-2,56	Afu3g14940	Elastase inhibitor AFUEI	aeiA
-2,68	Afu6g01870	Putative uncharacterized protein	N/A
-2,70	Afu6g11840	Sodium bile acid symporter family protein	N/A
-2,70	Afu6g00430	IgE-binding protein	N/A
-2,76	Afu8g02000	Sorbitol/xylitol dehydrogenase; putative	N/A
		V162(R)-ISA)	
		Up-Regulated	
2logFC	gene	Description	
8,95	Afu3g12910	O-methyltransferase GliM-like; putative	hasC
6,59	Afu3g01960	Putative uncharacterized protein	N/A
6,58	Afu6g11850	Putative uncharacterized protein	N/A
6,25	Afu4g02720	GPI anchored glycosyl hydrolase; putative	N/A
6,14	Afu1g01175	Putative uncharacterized protein	N/A
5,41	Afu8g06070	Putative uncharacterized protein	N/A
5,28	Afu7g04570	Na/K ATPase alpha 1 subunit; putative	N/A
5,12	Afu7g04550	Serine/threonine protein kinase; putative	N/A
5,12	Afu4g01290	Glycosyl hydrolase family 75 chitosanase; hydrolyzes beta-1,4-glycosidic linkage of chitosan; repressed by exposure to artemisinin	csnB
5,07	Afu2g17930	Integral membrane protein	N/A
4,95	Afu3g12920	Nonribosomal peptide synthetase 5	nrps5
4,86	Afu3g12960	Cytochrome P450 monooxigenase GliC-like; putative	hasH
4,85	Afu8g04920	LEA domain protein	N/A
4,76	Afu6g14140	RTA1 domain protein; putative	N/A
4,61	Afu6g03490	Phenol 2-monooxygenase; putative	fmpF
4,55	Afu5g08730	Putative uncharacterized protein	N/A
4,50	Afu4g00280	Putative uncharacterized protein	N/A
4,46	Afu6g11840	Sodium bile acid symporter family protein	N/A
4,34	Afu4g01260	Mitochondrial chaperone ATPase (Bcs1); putative	bcs1
4,06	Afu3g10370	Putative uncharacterized protein	N/A
4,00	Afu5g01230	RTA1 domain protein; putative	N/A
3,98	Afu3g01500	Integral membrane protein	N/A
3,93	Afu3g12930	7-dimethylallyltryptophan synthase	hasE
3,90	Afu8g00610	Cell surface protein Mas1; putative	mas1
3,90	Afu3g00710	Allergen Asp F4-like; putative	N/A
		Down-Regulated	
-2,90	Afu3g15100	Integral membrane protein; putative	N/A
-3,02	Afu7g00910	OPT peptide transporter Mtd1; putative	Mtd1
-3,07	Afu8g01850	Phosphate-repressible phosphate permease; putative	N/A
-3,09	Afu4g00450	Putative uncharacterized protein	N/A
-3,11	Afu1g15180	Putative uncharacterized protein	N/A
-3,11	Afu3g03420	Nonribosomal peptide synthetase 4	sidD
-3,12	Afu6g13930	LovB-like polyketide synthase; putative	pyr2
-3,15	Afu6g03190	Putative uncharacterized protein	N/A
-3,16	Afu4g01560	MFS myo-inositol transporter; putative	N/A
-3,17	Afu4g00830	MFS peptide transporter; putative	N/A
-3,23	Afu7g00420	Putative uncharacterized protein	N/A
-3,25	Afu5g02860	Integral membrane protein; putative	N/A
-3,28	Afu5g02850	Putative uncharacterized protein	N/A
-3,29	Afu8g06090	Amino acid permease; putative	N/A
-3,34	Afu6g01870	Putative uncharacterized protein	N/A
-3,39	Afu1g04130	FG-GAP repeat protein; putative	N/A
-3,42	Afu8g06100	Integral membrane protein	N/A
-3,45	Afu1g17580	Xenobiotic compound monooxygenase; DszA family; putative	N/A
-3,47	Afu7g06150	Endoglucanase; putative	N/A
-3,73	Afu7g06140	Probable beta-glucosidase L	exg13
-4,11	Afu4g13850	TfdA family oxidoreductase; putative	N/A
-4,13	Afu3g03650	GNAT family acetyltransferase; putative	sidG
-4,53	Afu1g03352	Alpha-1;3-glucanase/mutanase; putative	N/A
-4,70	Afu3g03640	MFS siderochrome iron transporter MirB	MirB
-5,18	Afu7g00440	GABA permease; putative	N/A
V181(R^PAN^)-ISA			
Up-Regulated			
2logFC	gene	Description	
4,31	Afu7g00480	ABC multidrug transporter; putative	abcE
3,21	Afu3g03530	Nitroreductase family protein	N/A
3,09	Afu3g14750	Fungal specific transcription factor; putative	N/A
2,87	Afu5g02260	ABC multidrug transporter; putative	N/A
2,71	Afu6g08140	Cytochrome P450 monooxygenase; putative	N/A
2,52	Afu4g01440	Glutathione S-transferase family protein	N/A
2,49	Afu3g00560	Putative uncharacterized protein, Has domain(s) with predicted catalytic activity, molybdenum ion binding, pyridoxal phosphate binding activity (	N/A
2,37	Afu1g06480	Putative uncharacterized protein, adhesin	N/A
2,37	Afu5g10060	Cytochrome b5 reductase; putative	N/A
2,34	Afu6g02220	MFS toxin efflux pump; putative	N/A
2,25	Afu5g10050	Cytochrome P450 monooxygenase; putative	N/A
2,21	Afu7g00150	FAD-dependent monooxygenase; putative	nscC
2,21	Afu7g01720	3-hydroxymethyl-3-methylglutaryl-Coenzyme A lyase	N/A
2,19	Afu4g14705	Putative uncharacterized protein	N/A
2,18	Afu3g14630	Extracellular dioxygenase; putative, Has domain(s) with predicted catalytic activity, ferric iron binding, iron ion binding, oxidoreductase activity	N/A
2,14	Afu1g17220	Probable endopolygalacturonase AFUA_1G17220	N/A
2,12	Afu3g08950	Putative uncharacterized protein	N/A
2,09	Afu5g10040	C6 transcription factor; putative	N/A
2,08	Afu2g10240	NAD binding Rossmann fold oxidoreductase; putative	N/A
2,08	Afu6g03380	Putative uncharacterized protein	N/A
2,07	Afu3g00960	Putative uncharacterized protein	N/A
2,04	Afu6g14140	RTA1 domain protein; putative	N/A
1,99	Afu5g10070	3-hydroxyacyl-CoA dehydrogenase; putative	N/A
1,98	Afu7g00470	Putative uncharacterized protein, FungiDB: possible transcription factor	N/A
		Down-Regulated	
-1,09	Afu1g13550	Putative uncharacterized protein	N/A
-1,09	Afu3g14950	Multicopper oxidase; putative	N/A
-1,11	Afu4g08420	Putative uncharacterized protein	N/A
-1,11	Afu3g08990	Cell surface protein; putative	cspA
-1,12	Afu6g10660	ATP citrate lyase subunit (Acl); putatibe	aclA
-1,12	Afu8g01910	Histidine acid phosphatase; putative	N/A
-1,13	Afu3g07560	Enoyl-CoA hydratase/isomerase family protein	N/A
-1,14	Afu6g11310	Bifunctional pyrimidine biosynthesis protein (PyrABCN); putative	N/A
-1,15	Afu4g00860	Dehydrin-like protein	dprA
-1,16	Afu5g02500	Putative uncharacterized protein	N/A
-1,19	Afu4g03240	Cell wall serine-threonine-rich galactomannoprotein Mp1	mp1
-1,19	Afu3g07710	Nucleolin protein Nsr1; putative	N/A
-1,19	Afu3g03940	2;3-diketo-5-methylthio-1-phosphopentane phosphatase; putative	N/A
-1,25	Afu5g00790	ABC multidrug transporter; putative	N/A
-1,25	Afu7g06580	FAD/FMN-containing isoamyl alcohol oxidase MreA-like; putative	N/A
-1,31	Afu6g00510	NADP-dependent alcohol dehydrogenase	N/A
-1,32	Afu7g01730	Phosphatidylserine decarboxylase family protein	N/A
-1,32	Afu7g06350	Sodium/phosphate symporter; putative	phoE
-1,33	Afu2g00625	Putative uncharacterized protein	N/A
-1,34	Afu4g08400	Putative uncharacterized protein	N/A
-1,35	Afu1g04300	Putative uncharacterized protein	N/A
-1,35	Afu5g01630	MFS multidrug transporter; putative	N/A
-1,40	Afu5g14740	Fucose-specific lectin FleA	fleA
-1,42	Afu8g07160	Putative uncharacterized protein	N/A
-2,15	Afu8g05810	DUF1295 domain protein	N/A

## Data Availability

The sequences reported in this paper have been deposited in the NCBI Sequence Read Archive (SRA) database in a BioProject under accession number PRJNA894836.

## Supplementary Materials

The following supporting information can be downloaded at: https://www.mdpi.com/article/10.3390/jof9080807/s1, Figure S1: Colony morphology of *A. fumigatus* isolates used in this study, Reference isolate CEA10 was added for comparison, Isolates were grown for 4d at 37 °C on AMM; Table S1: Complete list of DEseq2 results; Table S2: List of the 50 DEGs with the highest or lowest log2FC for each isolate; Table S3: List of DEGs after DMSO treatment; Table S4: List of DEGs found after Venn analysis; Table S5: All 26 secondary metabolite clusters in A. fumigatus and corresponding log2FC values; Table S6—Analysis of base expression levels; Table S7: Gene ontology enrichment analysis.
