# Supplementary material for: The Transcriptome Response to Azole Compounds in Aspergillus fumigatus Shows Differential Gene Expression across Pathways Essential for Azole Resistance and Cell Survival
Source: J Fungi (Basel). 2023 Jul 30;9(8):807. doi: 10.3390/jof9080807 (PMC10455693; doi:10.3390/jof9080807)
Supplement: Supplementary file 1 [file jof-09-00807-s001.zip › Figure S1.pdf]

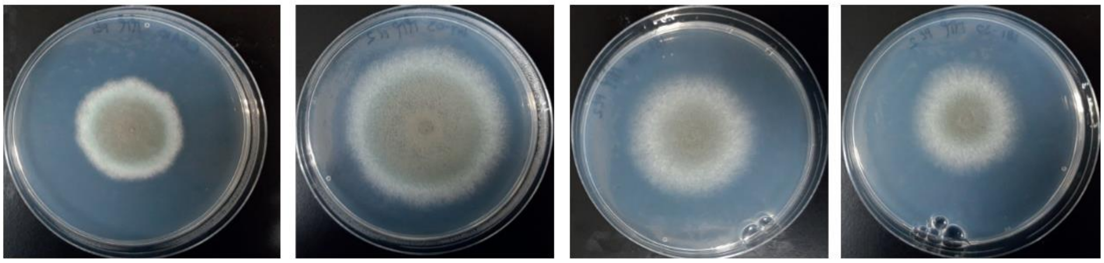

Figure S1 – Colony morphology of *A. fumigatus* isolates used in this study. Reference isolate CEA10 was added for comparison. Isolates were grown for 4d at 37°C on AMM.
