# Supplementary material for: The Transcriptome Response to Azole Compounds in Aspergillus fumigatus Shows Differential Gene Expression across Pathways Essential for Azole Resistance and Cell Survival
Source: J Fungi (Basel). 2023 Jul 30;9(8):807. doi: 10.3390/jof9080807 (PMC10455693; doi:10.3390/jof9080807)
Supplement: Supplementary file 1 [file jof-09-00807-s001.zip › Table S2.pdf]

Table S2 – Reads and coverage calculations. The samples have been analyzed by comparing each duplicate of azole treated samples to the 4 DMSO treated samples per isolate. The DMSO treated samples from isolate V147-03 conducted in another analysis were compared to an untreated sample of the same isolate.

Filename	Isolate	Treatment	Batch	# of Reads	Uniquely mapped reads	% of uniquely mapped reads	Coverage
V14703ITR01_R1.fastq.gz.Log.final	147-03	DMSO1	1	19052170	13.599.697	71.38%	70,83
V14703ITR02_R1.fastq.gz.Log.final	(S)	DMSO2	1	11904577	9.203.922	77.31%	47,94
S11-147D_R1.fq.gz.Log.final		DMSO1	2	26505688	25.138.574	94.84%	174,57
S12-147D_R1.fq.gz.Log.final		DMSO2	2	26505165	25.016.300	94.38%	173,72
V14703ITR50IC1_R1.fastq.gz.Log.final		ICZ1	1	9869332	6.235.921	63.18%	32,48
V14703ITR50IC2_R1.fastq.gz.Log.final		ICZ2	1	21676321	15.835.383	73.05%	82,48
S9-147I_R1.fq.gz.Log.final		ISA1	2	26530604	25.012.911	94.28%	173,70
S10-147I_R1.fq.gz.Log.final		ISA2	2	25443142	17.936.379	70.50%	124,56
V16206_1_48_R1.fastq.gz.Log.final	162-06	DMSO1	1	18954239	14.390.885	75.92%	74,95
V16206_2_48_R1.fastq.gz.Log.final	(R)	DMSO2	1	15869377	13.029.305	82.10%	67,86
Z11-162D_R1.fq.gz.Log.final		DMSO1	2	23622002	22.674.165	95.99%	157,46
Z12-162D_R1.fq.gz.Log.final		DMSO2	2	22560814	21.714.395	96.25%	150,79
V16206_1_48ITRA_R1.fastq.gz.Log.final		ICZ1	1	15045692	12.592.887	83.70%	65,59
V16206_2_48ITRA_R1.fastq.gz.Log.final		ICZ2	1	22093677	18.134.435	82.08%	94,45
R9-162I_R1.fq.gz.Log.final		ISA1	2	25136148	24.197.186	96.26%	168,04
Z10-162I_R1.fq.gz.Log.final		ISA2	2	26147354	24.874.645	95.13%	172,74
V18130ITR01_R1.fastq.gz.Log.final	181-30	DMSO1	1	17682151	14.359.777	81.21%	74,79
V18130ITR02_R1.fastq.gz.Log.final	(R^{PAN})	DMSO2	1	21619612	18.431.053	85.25%	96,00
R5-181D_R1.fq.gz.Log.final		DMSO1	2	26527964	25.617.320	96.57%	177,90
R6-181D_R1.fq.gz.Log.final		DMSO2	2	25313651	24.451.195	96.59%	169,80
V18130ITR50IC1_R1.fastq.gz.Log.final		ICZ1	1	11502052	9.416.369	81.87%	49,04
V18130ITR50IC2_R1.fastq.gz.Log.final		ICZ2	1	16487061	10.720.840	65.03%	55,84
R3-181I_R1.fq.gz.Log.final		ISA1	2	26404509	25.464.235	96.44%	176,83
R4-181I_R1.fq.gz.Log.final		ISA2	2	25290468	24.041.671	95.06%	166,96
	DMSO control isol						
D8-147_R1.fq.gz.Log.final	147-03	N/A	1	26340914	21846481	82.94%	151,71
D5-147D_R1.fq.gz.Log.final	(S)	DMSO1	1	25256166	24090060	95.38%	167,29
D6-147D_R1.fq.gz.Log.final		DMSO2	2	26241306	25009394	95.31%	173,68
